# The Na^+^/Ca^2+^, K^+^ exchanger 2 modulates mammalian cone phototransduction

**DOI:** 10.1038/srep32521

**Published:** 2016-09-01

**Authors:** Keisuke Sakurai, Frans Vinberg, Tian Wang, Jeannie Chen, Vladimir J. Kefalov

**Affiliations:** 1 Department of Ophthalmology and Visual Sciences, Washington University, Saint Louis, MO 63110, USA; 2 Zilkha Neurogenetic Institute, Department of Cell and Neurobiology & Department of Ophthalmology, Keck School of Medicine, University of Southern California, Los Angeles, CA 90033, USA.

## Abstract

Calcium ions (Ca^2+^) modulate the phototransduction cascade of vertebrate cone photoreceptors to tune gain, inactivation, and light adaptation. In darkness, the continuous current entering the cone outer segment through cGMP-gated (CNG) channels is carried in part by Ca^2+^, which is then extruded back to the extracellular space. The mechanism of Ca^2+^ extrusion from mammalian cones is not understood. The dominant view has been that the cone-specific isoform of the Na^+^/Ca^2+^, K^+^ exchanger, NCKX2, is responsible for removing Ca^2+^ from their outer segments. However, indirect evaluation of cone function in NCKX2-deficient (*Nckx2*^*−/−*^) mice by electroretinogram recordings revealed normal photopic b-wave responses. This unexpected result suggested that NCKX2 may not be involved in the Ca^2+^ homeostasis of mammalian cones. To address this controversy, we examined the expression of NCKX2 in mouse cones and performed transretinal recordings from *Nckx2*^*−/−*^ mice to determine the effect of NCKX2 deletion on cone function directly. We found that *Nckx2*^*−/−*^ cones exhibit compromised phototransduction inactivation, slower response recovery and delayed background adaptation. We conclude that NCKX2 is required for the maintenance of efficient Ca^2+^ extrusion from mouse cones. However, surprisingly, *Nckx2*^*−/−*^ cones adapted normally in steady background light, indicating the existence of additional Ca^2+^-extruding mechanisms in mammalian cones.

The detection of light in the vertebrate visual system and its transduction into an electric signal take place in the outer segments of rod and cone photoreceptors. Light triggers a phototransduction cascade that ultimately causes the closure of cyclic nucleotide-gated (CNG) channels in the photoreceptor outer segments^1^,^2^. The resulting reduction of the inward (dark) current causes hyperpolarization of the cell that alters the release of neurotransmitter at the retina’s first synapse between the photoreceptor and bipolar cells. In cone photoreceptors, about 30% of the inward dark current is carried by calcium ions^3^. In darkness, Ca^2+^ is at a steady state and its flow into the outer segment through the CNG channels is matched by its efflux. Upon light-induced closure of CNG channels, the Ca^2+^ inflow is reduced but its continuous extrusion out of the outer segment results in a decline in cytoplasmic Ca^2+^ in the outer segment^4^. This reduction in Ca^2+^ regulates several steps of the cone phototransduction cascade, which in turn modulate the recovery of the cone light response as well as background light adaptation^3^,^5^. In the absence of Ca^2+^ modulation, response recovery is greatly delayed and the photoreceptor cell saturates under low background light^6^,^7^. *In vitro* evidence indicates that the only mechanism of extrusion of Ca^2+^ out of the outer segments of vertebrate photoreceptors is by cell-specific Na^+^/Ca^2+^, K^+^ exchangers (NCKX)^8^, NCKX1 in rods and NCKX2 in cones^9^,^10^,^11^. However, a recent study of NCKX2-deficient (*Nckx2*^*−/−*^) mice found no alterations in cone number or in cone-driven bipolar cell responses assayed by photopic b-wave measurements from *in vivo* electroretinogram (ERG) recordings^12^. This unexpected result suggested that NCKX2 may not be involved in regulating the Ca^2+^ homeostasis of mammalian cones, leaving the mechanism of its extrusion from cone outer segments unknown. Here, we addressed this question by directly analyzing the physiological properties of NCKX2-deficient mouse cones in dark- and light-adapted conditions.

## Results

### NCKX2 is expressed in mouse cones

**Figure 1:**
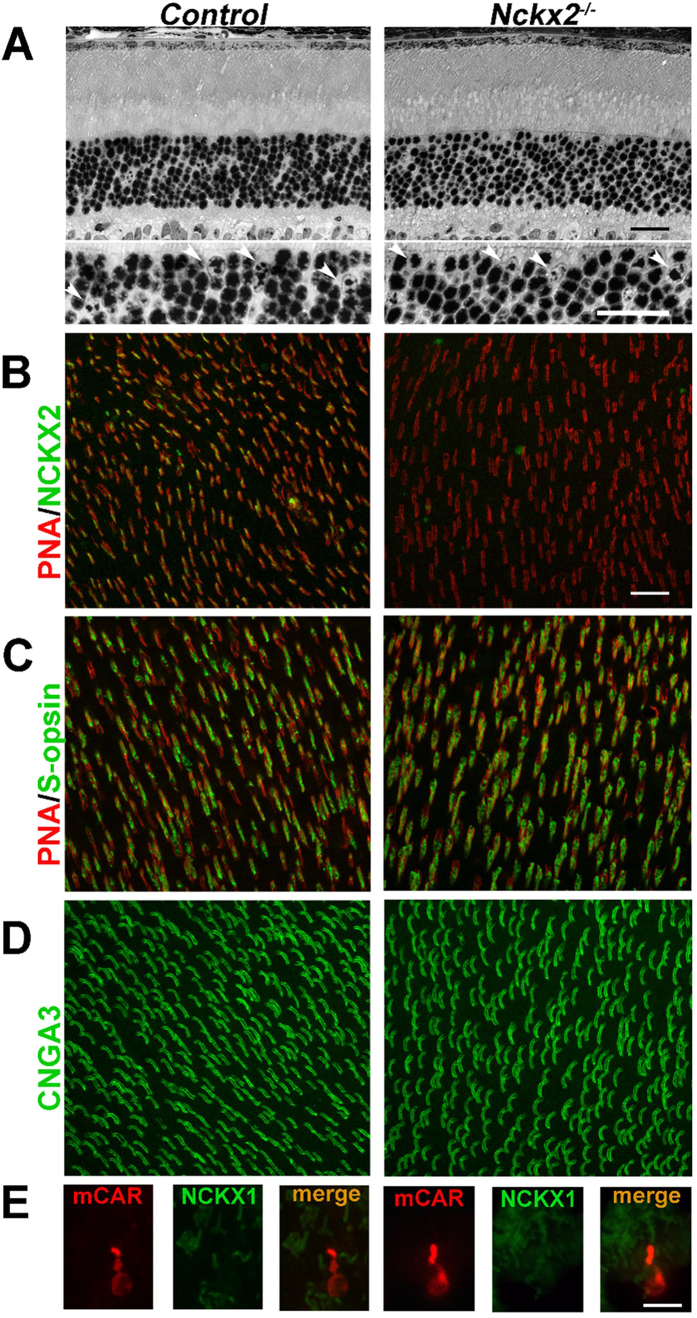
NCKX2 deficiency does not affect cone survival. (**A**) Light micrograph of retinal section from control (left) and NCKX2-deficient (right panel) mice show comparable retinal morphology. The cones, identifiable by the heterochromatic staining of cone nuclei (arrowheads) can be seen at higher magnification at the lower panel. (**B**) Whole mount staining of PNA and NCKX2 show cone-specific NCKX2 expression in the control retina (left panel) which is abolished in the *Nckx2*^*−/−*^ retina (right panel). (**C**) Inferior hemisphere of control (left panel) and *Nckx2*^*−/−*^ (right panel) retinas show a similar expression pattern of the short-wave cone opsin S-opsin. (**D**) Expression of cone-specific CNGA3 is also similar between control (left panel) and *Nckx2*^*−/−*^ (right panel) retinas. (**E**) Dissociated retinal cells were stained for cone marker (mCAR, cone arrestin, red) and NCKX1 (green). Scale bar = 20 μm. Both the control and the NCKX2-deficient mice were on *Gnat1*^*−/−*^ background which was used for subsequent cone recordings (Figs 3 and 4).

The presumptive cone-specific Na^+^/Ca^2+^, K^+^ exchanger, NCKX2, has been shown to be expressed in chicken and human cones by *in situ* hybridization^9^. However, the presence of a cone-specific exchanger and its localization in mouse cone photoreceptors has not been previously examined. Thus, before investigating the possible functional role of NCKX2 in mouse cones, we first sought to establish its expression there. Comparison of light micrographs of retinal sections from 3 month-old littermate control and NCKX2-deficient mice revealed comparable retinal morphology, with similar number of cone photoreceptors that are recognizable by the heterochromatic staining pattern of their nuclei (Fig. 1A bottom panel; arrowheads). Notably, co-labeling of retinal flat mounts with the cone-specific Peanut Agglutinin (PNA) and with NCKX2 antibody revealed expression of NCKX2 selectively in the cone outer segments of control (Fig. 1B; left panel) but not *Nckx2*^*−/−*^ (Fig. 1B, right panel) retinas. This result demonstrates the expression of NCKX2 in mouse cone photoreceptors and its successful deletion in *Nckx2*^*−/−*^ mice. Consistent with the retinal sections results above, the number of PNA-labeled photoreceptor cells in retinal flat mounts was comparable in control and NCKX2-deficient retinas (Fig. 1B), as were the expression of cone S-opsin at the inferior retina (Fig. 1C) and the cone cyclic nucleotide-gated (CNG) channel A3 subunit (Fig. 1D). Quantification showed similar numbers of PNA-positive cones in C57Bl/6, *Gnat1*^*−/−*^ control, and NCKX2-deficient *Gnat1*^*−/−*^ mice (340 ± 60, 360 ± 40, 350 ± 50, mean ± SD of cones in 0.3 mm^2^, n ≥ 7; p = 0.65 by one-way analysis of variance). To investigate whether ablation of NCKX2 expression led to compensatory changes in expression of other known retinal exchangers in cones, we examined expression of NCKX1 in dissociated retinal cells. NCKX1 is known to be specifically expressed in rods^13^. Indeed, in dissociated photoreceptors from the control sample, NCKX1 immunoreactivity (green, Fig. 1E) could be seen in the rod outer segment, but not in cones, labeled with cone arrestin (Fig. 1E, mCAR, red). A similar staining pattern was observed in the NCKX2-deficient *Gnat1*^*−/−*^ sample indicating a lack of compensatory changes in the expression of NCKX1. Having established that NCKX2 is expressed in mouse cone outer segments and that its deletion does not affect cone survival, we next investigated the functional role of this exchanger in cones.

### NCKX2 modulates the kinetics of mouse cone flash responses

**Figure 2:**
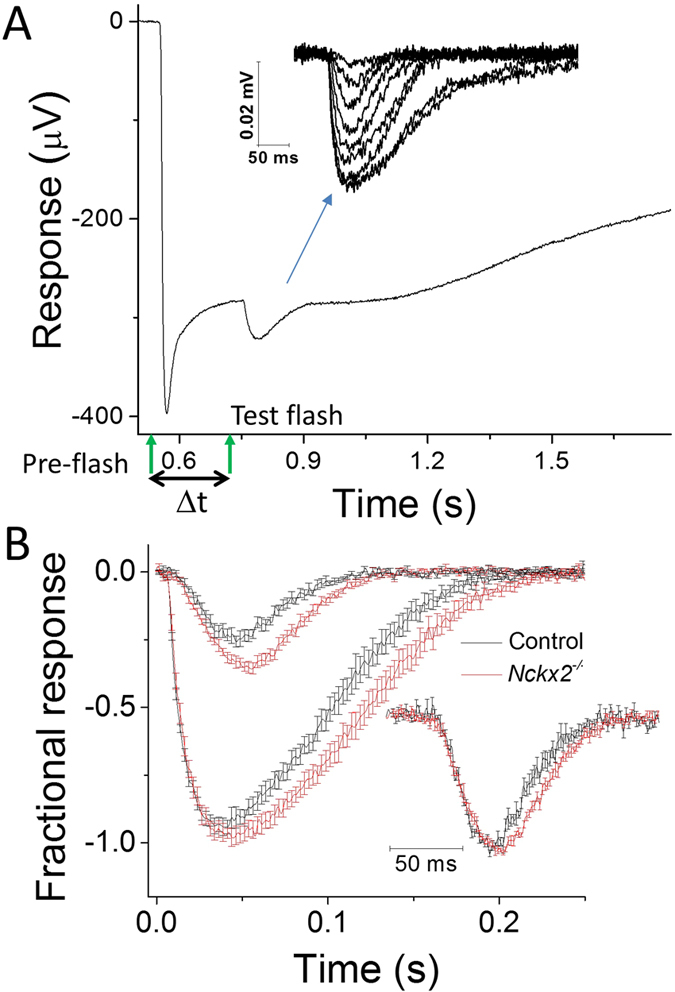
Double flash-isolated cone responses from *Nckx2*^*−/−*^ and littermate control mice. (**A**) Double flash method to isolate cone light responses in *ex vivo* retina preparation. Pre-flash delivering 12,000 photons (530 nm) μm^−2^ (left green arrow) was followed by a test flash (right green arrow) delivered while the rods were still saturated. Inter-stimulus interval Δt was 200 ms. Inset: representative cone response family to test flashes ranging from 565–732,000 photons μm^−2^. (**B**) Population averaged dim flash responses (I = 1,600 photons μm^−2^) and saturated bright flash responses (I = 183,000 photons μm^−2^) normalized with R_max_ in control (black, n = 6) and *Nckx2*^*−/−*^ (red, n = 8) retinas, respectively. The inset shows normalized dim flash responses from control (black) and *Nckx2*^*−/−*^ (red) retinas. Error bars show SEM.

A disruption of Ca^2+^ extrusion from the outer segments of cone photoreceptors would be expected to delay the Ca^2+^-mediated negative feedback on the cone phototransduction cascade and result in abnormally slow response recovery. However, a recent study found that the deletion of the putative cone-specific Na^+^/Ca^2+^, K^+^ exchanger, NCKX2, did not alter the b-wave recorded in photopic (cone-driven) light conditions^12^. The b-wave is generated by the activation of ON bipolar cells^14^,^15^, and provides, therefore, only an indirect evaluation of upstream cone function. On the other hand, the a-wave directly measures the summed response from photoreceptor cells. However, the cone a-wave is difficult to observe with *in vivo* ERG recordings, particularly at subsaturating flash intensities^16^,^17^, due to the small number of cones in mouse retina. To overcome these obstacles, we turned to *ex vivo* ERG recordings which allowed us to obtain flash responses from an isolated retina (see Methods for details). This methodology provides excellent signal-to-noise ratio which allows quantitative analysis of dim flash responses^16^,^18^. Furthermore, such *ex vivo* recordings allow pharmacological block of the b-wave to unmask the full time course of the photoreceptor-driven a-wave. Thus, we began our analysis of the role of NCKX2 by comparing the *ex vivo* a-wave photoresponse in *Nckx2*^*−/−*^ mice and their littermate controls. To isolate the a-wave from the downstream components of the retinal response, including the b-wave, we perfused the retina with a cocktail of synaptic blockers (see Methods for details). The cone component of the mouse retina response is typically overwhelmed by the much larger rod-driven response (Fig. 2A, first flash). Thus, we isolated the cone component of the retina response by suppressing the rod component with a saturating pre-flash. This allowed us to obtain cone responses to a range of test flash intensities, from threshold to saturation (Fig. 2A, second flash; inset). Comparison of the responses from cones in control and *Nckx2*^*−/−*^ retinas revealed that the deletion of NCKX2 did not affect the early rising phase but caused a notable delay in the response recovery observable throughout the range of flash responses, from threshold to saturation (Fig. 2B). Together, these results could be explained by slower negative feedback on the cone phototransduction cascade due to a delayed extrusion of Ca^2+^ from the cone outer segments of NCKX2-deficient cones. However, the relatively complicated protocols required for the isolation of the cone component of the retinal response prevented us from investigating in a rigorous way the effect of NCKX2 deletion of cone function under dark-adapted conditions and, even more so, in background light. In addition, potential differences in the adaptation properties of control and NCKX2-deficient cones could have contributed to the observed “paired-flash” phenotype.

**Figure 3:**
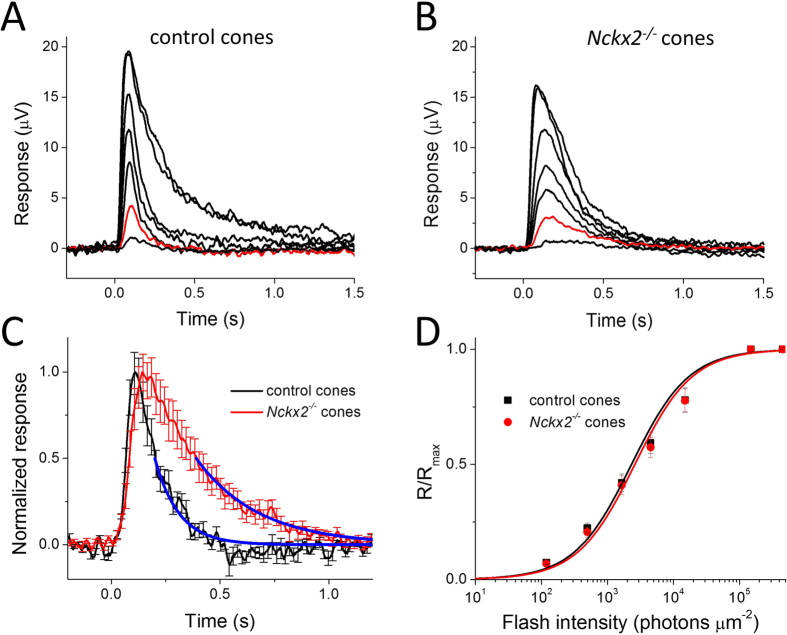
Flash response families of dark adapted control (**A**) and *Nckx2*^*−/−*^ cones (**B**) from transretinal recordings from Gnat1-deficient mice. Cone responses were evoked by a series of 500-nm test flashes (10 ms in duration) with intensities (photons μm^−2^) 1.2 × 10^2^, 5.0 × 10^2^, 1.6 × 10^3^, 4.5 × 10^3^, and 1.5 × 10^4^. The two saturated responses in each case were triggered by white flashes differing in intensity by ~3-fold. The red traces indicate responses to a 500 photons μm^−2^ test flash. (**C**) Population-averaged normalized dim flash responses of control (black) and *Nckx2*^*−/−*^ (red) cones. The single exponential fits to the second half of the response recovery (blue lines) give recovery time constants of 111 ms and 287 ms in control and *Nckx2*^*−/−*^ cones, respectively. Error bars show SEM. (**D**) Intensity-response relations of cone transretinal responses from control (black squares) and *Nckx2*^*−/−*^ (red squares) retinas. The solid curves represent the corresponding intensity-response functions (Eqn. (1)) with *I*_*o*_ of 2,300 photons μm^−2^ and 2,600 photons μm^−2^, respectively.

**Figure 4:**
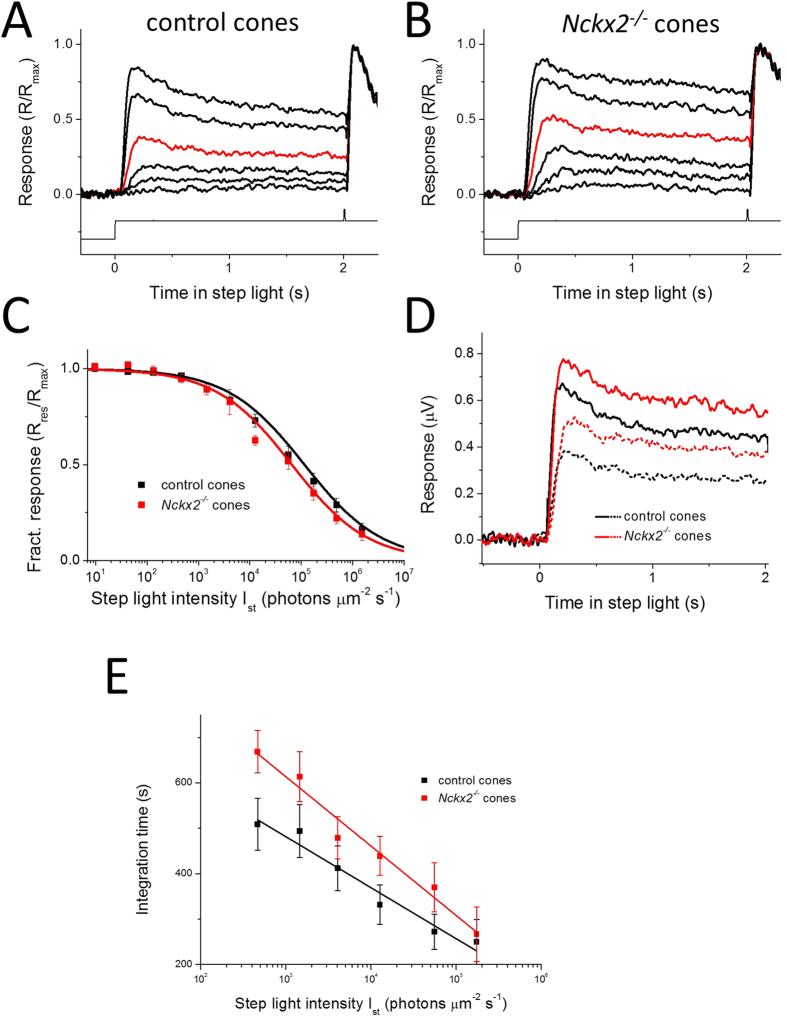
Light adaptation of control and *Nckx2*^*−/−*^ cones. Responses were evoked by step of 500 nm light (at t = 0 s) followed by bright saturating test flash in control (**A**) and *Nckx2*^*−/−*^ (**B**) cones from Gnat1-deficient mice. Intensity of the step lights was 470, 1.5 × 10^3^, 4.1 × 10^3^, 1.3 × 10^4^, 5.6 × 10^4^, 1.7 × 10^5^ photons μm^−2^ s^−1^. The red traces indicate responses to a 13,000 photons μm^−2^ step of light. (**C**) Residual response amplitude 2 s after onset of step light in control (black, n = 8) and *Nckx2*^*−/−*^ (red, n = 7) cones. Fractional residual response, *R*_*res*_*/R*_*max*_, was estimated as the amplitude of the residual saturated response *R*_*res*_ evoked 2 s after onset of the background exposure, normalized to the maximal response in darkness *R*_*max*_. Solid curves indicate the fitting function of Eqn. (2) with *I*_*b*_ of 1.2 × 10^5^ and 6.5 × 10^4^ photons μm^−2^ s^−1^ and *k* of 0.58 and 0.59 for control and *Nckx2*^*−/−*^ cones, respectively. (**D**) Comparison of the responses of control (black) and *Nckx2*^*−/−*^ (red) cones to backgrounds of 4.1 × 10^3^ photons μm^−2^ s^−1^ (dashed lines) and 1.7 × 10^5^ photons μm^−2^ s^−1^ (solid lines), replotted from (**A,B**). (**E**) Integration times of saturated responses to identical test flashes elicited in backgrounds of increasing intensity for control (n = 8) and *Nckx2*^*−/−*^ (n = 7) cones. The test flashes were delivered 2 s after the onset of the background as shown in (**A,B**), respectively. Error bars show SEM. Linear fits to each data set reveal the convergence of the response kinetics for the two genotypes with increasing background light intensities.

To address these technical issues, we used an alternative, genetic approach for isolating the cone response: by crossing the *Nckx2*^*−/−*^ mice with mice lacking the rod transducin α subunit (Gnat1, a key component of the rod phototransduction cascade). The deletion of Gnat1 prevents the rods from generating photoresponses while preserving normal retinal morphology^19^,^20^. Thus, after blocking the b-wave pharmacologically, the photoresponse of Gnat1-deficient retinas will be generated exclusively by cones at all light levels, even under dark-adapted conditions^21^,^22^. Comparison of cone flash responses from control (*Gnat1*^*−/−*^) mice (Fig. 3A) and NCKX2-deficient (*Gnat1*^*−/−*^*Nckx2*^*−/−*^) mice (Fig. 3B) confirmed that the deletion of NCKX2 substantially delays the cone response recovery: the time to peak, integration time, and the recovery time constant of the cone dim flash response were all significantly increased in the absence of NCKX2 (Fig. 3C; Table 1). Together, these results indicate that extrusion of Ca^2+^ via NCKX2 plays a key role in the timely inactivation of the cone phototransduction cascade within the time course of the cone flash response (~1 s). Notably, the deletion of NCKX2 led to a subtle but statistically significant decrease of the maximal response amplitude (R_max_) but did not change the sensitivity of cones to light flashes (I_0_) (Fig. 3D, Table 1). The late rising phase of the cone flash response appeared slightly delayed (Fig. 3C). However, this effect was not observed in our pair-flash responses (Fig. 2B) or in responses to step of light (Fig. 4D), indicating that deletion of NCKX2 produced only a minor, if any, effect on the activation of the phototransduction cascade. The largely normal cone function in NCKX2-deficient cones is consistent with the lack of detectable cone loss in the absence of NCKX2 (Fig. 1).

### NCKX2 modulates mouse cone light adaptation

Disrupting the timely extrusion of Ca^2+^ from the cone outer segments would also be expected to delay sensitivity adjustment during light adaptation. To examine this issue, we exposed control (*Gnat1*^*−/−*^) retinas (Fig. 4A) and NCKX2-deficient (*Gnat1*^*−/−*^*Nckx2*^*−/−*^) retinas (Fig. 4B) to 2-seconds steps of steady light of increasing intensity. As expected^6^, in control retinas the step of light produced an initial peak response followed by partial relaxation over time, mediated by the adaptation of the cones to steady background light (Fig. 4A). This adaptation was particularly pronounced for brighter backgrounds. Notably, similar relaxation in the step response amplitude and the corresponding *R/R*_*max*_ ratio could also be observed for NCKX2-deficient cones (Fig. 4B). This result demonstrates that *Nckx2*^*−/−*^ cones were able to undergo background adaptation despite the lack of NCKX2-dependent extrusion of Ca^2+^ from their outer segments. Indeed, the fractional response suppression at steady state, measured at the end of the 2 s background light exposure, was comparable in control and NCKX2-deficient cones (Fig. 4C). However, consistent with slower extrusion of Ca^2+^ from cone outer segments, the onset of response recovery, driven by the calcium-mediated negative feedback, was delayed in *Nckx2*^*−/−*^ cones compared to controls (Fig. 4D) in steady background light of both bright (solid lines) and intermediate (dashed lines) intensity. Comparison of the integration times of saturated responses elicited 2 s after the onset of background light from control and *Nckx2*^*−/−*^ cones revealed that, for dim backgrounds, flash responses were substantially slower in the absence of NCKX2 (Fig. 4E). This result is consistent with our findings from dark-adapted cones (Figs 2 and 3). However, the difference in kinetics between control and NCKX2-deficient responses gradually declined with increasing backgrounds, indicating progressive calcium reduction and robust acceleration of the phototransduction shutoff in *Nckx2*^*−/−*^ cones. Together, these results suggest that rapid Ca^2+^ extrusion by NCKX2 is required for the timely light adaptation of cones. However, the deletion of NCKX2 delayed, but did not block, the Ca^2+^-mediated light adaptation in mouse cones (Fig. 4C). These results suggest the existence of additional Ca^2+^ extrusion routes from the cone outer segment compartment.

## Discussion

The mechanism of Ca^2+^ extrusion from mouse cone outer segments by the putative NCKX2-dependent pathway was put into question due to the lack of cone-driven b-wave ERG phenotype in *Nckx2*^*−/−*^ mice^12^. Here we have resolved this issue by examining the expression of NCKX2 in mouse cones and directly recording the flash responses of *Nckx2*^*−/−*^ cones. Our results demonstrate that NCKX2 is expressed selectively in the outer segments of mouse cone photoreceptors and that its deletion in *Nckx2*^*−/−*^ mice does not cause detectable cone degeneration (Fig. 1). The better resolution of our transretinal recordings compared to the previous *in vivo* ERG study of the role of NCKX2 in cone function^12^ allowed us to show clearly that its deletion affects the flash responses of dark-adapted mouse cones as well as their light adaptation. Specifically, we found that the deletion of NCKX2 from mouse cones slows down the recovery of their light responses (Figs 2 and 3). This finding is consistent with NCKX2-mediated Ca^2+^ extrusion being an integral component of the rapid feedback to the cone phototransduction cascade and its timely inactivation. Such a conclusion is also supported by the delayed onset of cone background adaptation in NCKX2-deficient mice (Fig. 4), indicative of their slowed Ca^2+^ extrusion.

Block of the major Ca^2+^-mediated negative feedback on cone phototransduction by deleting the two guanylyl cyclase activating proteins (GCAPs) results in several-fold larger cone single photon response and dramatically delayed response recovery^21^. Thus, if NCKX2 is the dominant mechanism for extruding Ca^2+^ from cone outer segments, it would be expected that its deletion would largely delay the onset of the Ca^2+^-mediated feedback on GCAPs so that the physiological phenotype in *Nckx2*^*−/−*^ cones would be comparable to that in GCAPs knockout cones. However, we found that the deletion of NCKX2 in mouse cones produces only a slight delay in the response recovery without affecting substantially the amplitude of the single photon response (Fig. 2). Thus, the GCAPs-mediated negative feedback on cone phototransduction is still largely functioning in NCKX2-deficient cones, indicating that the deletion of this exchanger does not affect dramatically the extrusion of Ca^2+^ from mouse cones. Consistent with this notion, we also found that given sufficient time (~2 s), the background light response amplitude of *Nckx2*^*−/−*^ cones reaches steady state comparable to that of control cones (Fig. 4C). Together, these surprising results clearly indicate that extrusion of Ca^2+^ still occurs in the absence of NCKX2. The observation of normal cone survival in *Nckx2*^*−/−*^ retina also supports the existence of additional, yet to be identified Ca^2+^ extrusion pathway(s) for the maintenance of Ca^2+^ homeostasis in cones, because complete block of Ca^2+^ extrusion would be expected to lead to abnormally high and toxic Ca^2+^ levels^23^,^24^.

Interestingly, we recently found that disruption of NCKX1, the rod-specific exchanger isoform, leads to down-regulation of the rod CNG channel and greatly diminished maximal light responses in *Nckx1*^*−/−*^ rods^13^. This regulation of transduction channel expression by the exchanger may serve as an adaptive mechanism in rods to prevent Ca^2+^ entry when Ca^2+^ extrusion is disrupted. In contrast, the maximum light response is comparable between control and NCKX2-deficient cones, indicating a normal complement and function of cone CNG channels (Fig. 1D, also compare Fig. 3A,B). This difference in the phenotype between NCKX-deficient rods and cones is not yet fully understood, but may be related to the biochemically characterized direct interaction between NCKX1 and the rod CNG channel^25^ but not between NCKX2 and the cone CNG channel^26^. Therefore a normal route of Ca^2+^ entry through functional CNG channels still exists for *Nckx2*^*−/−*^ cones. This finding also supports the existence of efficient Ca^2+^ extrusion mechanism(s) in NCKX2-deficient cones.

Based on these results, the emerging model is that NCKX2 plays an important role in the rapid modulation of the phototransduction cascade at the sub-second time scale of the mouse cone response. Consistent with this concept, the rapid onset of cone light adaptation is delayed in the absence of NCKX2 (Fig. 4D). However, on a longer time scale (~2 s), extrusion of Ca^2+^ still appears to proceed despite the removal of NCKX2. Thus, our results reveal the existence of a novel, yet to be identified NCKX2-independent cone Ca^2+^ extrusion pathway. One possibility is that another exchanger is also expressed in the outer segments of cones. We show that the rod-specific exchanger NCKX1 is not expressed in mouse cones^13^, or in NCKX2-deficient cones (Fig. 1E) ruling out NCKX1 as a possibility. The lack of isoform-specific exchanger blockers^27^ makes addressing this issue possible only by molecular and genetic tools in the future. Alternatively, Ca^2+^ in the outer segment could move down the concentration gradient into the inner segment through the connecting cilium and then be taken up by intracellular stores, such as mitochondria^28^ and the endoplasmic reticulum^29^, or extruded by mechanisms in the inner segment plasma membrane. Consistent with this notion, expression of the Na^+^/Ca^2+^ exchanger NCX1 has been reported in the cone inner segments^30^. However, such a mechanism would be expected to be relatively slow and play a role more in the long-term regulation of calcium rather than in its modulation within the time course of the cone flash response. Finally, the plasma membrane Ca^2+^ ATPase (PMCA) expressed in cones could also be involved in the regulation their Ca^2+^ homeostasis^31^. Future studies should help resolve this issue.

## Methods

### Animals

The use of mice in these experiments was in accordance with the guidelines established by the animal care and use guidelines of our respective universities and all experimental protocols were approved by the Animal Studies Committees of Washington University and the University of Southern California. *Nckx2*^*−/−*^ mice were kindly provided by Jonathan Lytton (Li *et al.*^12^). To facilitate cone recordings, control and *Nckx2*^*−/−*^ mice used in all experiments except those shown in Fig. 2 were on the background of *Gnat1*^*−/−*^ mice (Calvert *et al.*, 2000) which produce no rod photoresponses. Experiments were done with young adult mice (2–3 months-old) from either sex.

### Light microscopy of retinal morphology

Eyecups were marked at the superior pole by cauterization. Eyes were placed in fixative for 5 min (2.5% glutaraldehyde, 2% formaldehyde in 0.1M cacodylate buffer, pH 7.2). The lens and cornea were dissected out, and the remaining eyecup further fixed overnight. Fixed tissues were rinsed in 0.1M cacodylate buffer, dehydrated by graded ethanol washes and embedded into epoxy resin. Tissues were sectioned at 1 μm thickness along the vertical meridian and images were acquired on a Zeiss Axioplan2 microscope (Carl Zeiss, Oberkochen, Germany) using a 63X objective.

### Immunocytochemistry

Eyes were enucleated and fixed in 4% formaldehyde in phosphate buffered saline (PBS) for 1 hour. The cornea and lens were then removed and the remaining eyecup was placed back in 4% formaldehyde in PBS for another 2 hours. After this second fixation step the retinal pigmented epithelium was carefully peeled away from the neuroretina. The remaining tissue was further fixed in 4% formaldehyde in PBS for 1 hour. The fixed tissue was then washed 3 times in PBS and blocked with 2% bovine serum albumin, 2% goat serum, 0.3% Triton X-100 in PBS for 1 hour. Neuroretinas were then incubated overnight at 4 °C with the following primary antibodies: rabbit NCKX2 N2F (1:100; ref. 12), mouse s-opsin 95392 (1:200; ref. 20), and rabbit CNGA3 (1:80; Alomone Labs, Jerusalem, Israel). The whole mounts were rinsed and incubated with a fluorescein-labeled secondary antibody (1:400; Vector Laboratories, Burlingame, California, USA) and PNA-Rhodamine (1:400; Vector Laboratories) for 1 hour. Four cuts were made at the edge of the retina for flat mounting and placed on slides for imaging using Zeiss LSM5 Pascal confocal microscope using identical settings on control and *Nckx2*^*−/−*^ retinas. For the dissociated cells, retinas were chopped in PBS. The cell suspension was placed on gelatin-coated glass slides and centrifuged for 3 min at 500 rpm. The cells were fixed for 5 min in 4% formaldehyde in PBS, washed, and processed for immunofluorescence as described for frozen sections. The primary antibodies used were LUMI-J that binds cone arrestin^32^ and 8H6 that binds NCKX1^13^.

### Cone quantification

Confocal images of whole-mount retinas with PNA stained cones were acquired using a 40X objective. Each image was analyzed using the ImageJ freeware (https://imagej.nih.gov/ij/). First the cones were highlighted using the threshold function. This was followed by processing the image into binary form. The cones were then counted using the “analyze particles” function. A minimum of 7 fields from two retinas was used to quantify the number of cones.

### Transretinal ERG recordings

Transretinal recordings from *Gnat1*^*−/−*^ control and *Nckx2*^*−/−*^ *Gnat1*^*−/−*^ mice were performed as described previously^21^. Briefly, after euthanasia, eyes of dark-adapted mice were hemisected and retinas were isolated in cold Ringer solution (112 mM NaCl, 3.6 mM KCl, 2.4 mM MgCl_2_, 1.2 mM CaCl_2_, 10 mM HEPES, 10 mM glucose pH 7.4). Half of the dorsal retina was transferred to the recording chamber on filter paper with the photoreceptor side up. To remove the electrical components of higher-order neurons, 2 mM L-glutamate was added to the perfusion solution (Ringer solution plus 3 mM Na_2_-succinate, 0.5 mM Na-glutamate, 20 mM NaHCO_3_, and equilibrated with 95% O_2_-5% CO_2_ at 34 °C). The electrode solution under the retina also contained 10 mM BaCl_2_ to suppress the glial component of the response. All solution chemicals were purchased from Sigma-Aldrich, Saint Louis, Missouri, USA). Responses were amplified by a differential amplifier (DP-311; Warner Instruments, Hamden, Connecticut, USA), low-pass filtered at 30 Hz, and digitized at 1 kHz. For light stimulus, 500-nm flash light or white light (>410 nm) was delivered after attenuation with neutral density filters. Sensitivity of flash response was estimated by fitting the following equation:


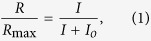


where *R* is response amplitude, *R*_*max*_ is maximal response amplitude, *I* is flash intensity, and *I*_*o*_ is the flash intensity required to produce half-maximal response. Time to peak was estimated as the time from the onset of the flash to the peak of a response from the linear range (<30% R_max_). From the same responses, integration time was estimated as the integral of the dim flash response normalized to its peak amplitude, and recovery time constant was estimated as the single exponential fit to the late phase (past 50%) of the response recovery. The residual response in background illumination, *R*_*res,*_ was measured from a saturating flash response at the end of the 2 s step light exposure and *R*_*res*_*/R*_*max*_ was fit to the Hill equation:


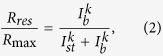


where *I*_*st*_ is step light intensity, *I*_*b*_ is the step light intensity to produce half-maximal response amplitude, and *k* is the Hill coefficient.

Cone responses in WT and their *Nckx2*^*−/−*^ littermate control mice expressing rod transducin α were isolated by using double flash technique as described previously^33^ by using transretinal ERG technique described above. Perfusion solution in these experiments was bicarbonate buffered Locke’s solution bubbled with 5% CO_2_ carbogen at 37 °C (same as in *Gnat1*^*−/−*^ experiments, see above) supplemented with 40 μM DL-AP4 (#0101; Tocris Biosciences, Bristol, United Kingdom), 2 mM L-Aspartate and 100 μM BaCl_2_ to isolate the photoreceptor component of transretinal ERG signal. Data was sampled at 10 kHz and low-pass filtered at 300 Hz (8-pole Bessel filter). We used pre-flash (flash length 1 ms) of 12,000 photons (530 nm) μm^−2^ to saturate rods and test flashes from 565 to 732,000 photons μm^−2^ (flash length 1 ms except for the brightest flashes up to 4 ms) with inter-stimulus interval of 200 ms to elicit pure cone responses without contribution from rod photoreceptors that remained saturated at that time.

By selecting the dorsal, M-opsin-rich part of the retina for recordings and by using longer wavelength test flashes (500 nm and 565 nm), we focused our studies on cones expressing M-opsin. Considering that in the mouse retina most cones express M-opsin or co-express M-opsin and S-opsin^34^, and that true S-cones are rare (3–5% of all cones^35^), our findings would apply to the bulk of mouse cone photoreceptors.

### Statistics

Two-tailed unpaired Student’s t-test was used to test for the significance of difference in the mean values of two sample groups. P-values of <0.05 were considered to be statistically significant.

## Additional Information

**How to cite this article**: Sakurai, K. *et al.* The Na^+^/Ca^2+^, K^+^ exchanger 2 modulates mammalian cone phototransduction. *Sci. Rep.* **6**, 32521; doi: 10.1038/srep32521 (2016).

## Figures and Tables

**Table 1:** Cone response parameters from transretinal recordings.

	control (n = 14)	*Nckx2*^*−/−*^ (n = 15)	p-value
Dark-adapted response			
Time to peak (ms)	110 ± 4	171 ± 6	0.001
Integration time (ms)	203 ± 21	391 ± 28	0.001
Recovery time constant (ms)	111 ± 14	287 ± 30	0.001
R_max_ (μV)	15 ± 1	12 ± 1	0.01
I_o_ (photons μm^−2^)	2,310 ± 320	2,650 ± 340	0.48
Light-adapted response			
I_b_ (photons μm^−2^)	115,000 ± 39,000	66,000 ± 17,000	0.27
Hill coefficient	0.58 ± 0.01	0.59 ± 0.03	0.96

The kinetic parameters were measured from dim flash responses with amplitude less than 20% *R*_*max*_. The step light intensity *I*_*b*_ that produced half-maximal response amplitude and the Hill coefficient *k* were obtained from the light adaptation experiments shown in Fig. 4C. Statistical analyses were made with Student’s t-test. Values given as mean ± SEM. All recordings were from Gnat1-deficient mice.
